# Perturbation-evoked cortical responses and altered causal information flow reflect more effortful but less efficient postural control in patients after ACLR

**DOI:** 10.1038/s41598-026-55383-2

**Published:** 2026-06-10

**Authors:** Tim Lehmann, Gjergji Cobani, Romina Müller, Jochen Baumeister

**Affiliations:** 1 Exercise Science & Neuroscience, Department of Exercise & Health, Faculty of Science, Paderborn University, Paderborn, Germany; 2 Biomedical Engineering and Radiation Physics, Department of Diagnostics and Intervention, Umeå University, Umeå, Sweden

**Keywords:** Neurology, Neuroscience

## Abstract

Effective responses to sudden mechanical perturbations require coordinated neural processes for generating rapid, situation-specific postural reactions. Sensorimotor impairments following anterior cruciate ligament reconstruction (ACLR) may disrupt this coordination and contribute to inefficient postural control strategies. Therefore, the present study investigated perturbation-evoked behavioral and cortical dynamics in individuals after ACLR compared with asymptomatic controls. Seventeen athletes after ACLR (8 female, 23.6 ± 3.7 years) and thirteen controls (4 female, 25.9 ± 4.2 years) underwent 100 unpredictable platform translations in bipedal stance. Postural responses were examined using statistical parametric mapping of anterior–posterior hip acceleration. Cortical dynamics were assessed via mobile electroencephalography by quantifying perturbation-evoked potentials (PEP N1) and effective connectivity derived from renormalized partial directed coherence. Compared to controls, the ACLR group exhibited significantly higher hip acceleration during early voluntary adjustment and late re-stabilization phases of the postural response. Due to the different approaches of group-level comparisons, 20 participants (11 ACLR / 9 CON) showing characteristic event-related potentials were used for the PEP analysis, whereas 30 participants (17 ACLR / 13 CON) were eligible for the effective connectivity analysis utilizing probabilistic dipole densities. N1 amplitudes of the PEPs were significantly higher in the ACLR group, whereas fronto-central information flow was significantly lower early after perturbation, but significantly higher during re-stabilization. These results suggest that postural responses after ACLR may rely on increased cortical activation and altered dynamics within a fronto-central network,  pointing at greater voluntary control and reduced automatization of sensorimotor processes, likely predisposing individuals after ACLR to an elevated risk of dysfunction and re-injury.

## Introduction

Adequate responses to sudden perturbations of human upright stance inherently require a distributed neural network to instantaneously initiate protective motor actions for maintaining body alignment and lower limb joint stability^1–3^. An effective postural response therefore necessitates an intact sensorimotor system to rapidly perceive afferent sensory information from different modalities to generate situation-specific motor commands for appropriately controlling joint alignment and maintaining the center of mass (CoM) within the base of support^2–4^. With this, postural control also constitutes a crucial determinant for the quality of complex full-body movement through predictable and unpredictable, as well as stable and dynamic environments, being closely related to measures of athletic performance^3,5^. However, in case of sensorimotor deficiencies caused by trauma, such as those observed after anterior cruciate ligament reconstruction (ACLR), insufficient afferent information can negatively affect postural control strategies and ultimately manifest as inaccurate postural reactions^6–8^. Consequently, patients after ACLR exhibit altered compensatory and reactive postural responses to unpredictable whole-body perturbations, which may pose an increased risk of re-injury for these individuals^9–12^.

In this regard, the articular structures damaged by ACL injury and surgical reconstruction not only limit motion within the natural range of a joint, but normally also function as an important source of sensory information^13^. Induced by articular motion and subsequent mechanical deformation within or around the articulation, mechanoreceptors, muscle spindles and Golgi tendon organs collectively generate the somatosensory neural impulses necessary to mediate protective motor actions in higher levels of the central nervous system for the regulation of posture and joint stability^3^. In situations of sudden mechanically evoked postural instability, studies utilizing non-invasive electroencephalography (EEG) have shown that somatosensory, visual or vestibular disturbances elicit an early multicomponent vertex potential with a characteristic polarity, morphology, and peak latency in fronto-central brain areas. In the context of postural control, this time-locked electrophysiological response is typically referred to as the perturbation-evoked potential (PEP) and might represent an extralemniscal (non-specific) cortical response that is engaged by rapid stimulus changes and particularly sensitive to salient or potentially threatening sensory stimuli^1,14,15^. The most prominent and robust component of the PEP is a large negative deflection (N1) with a peak amplitude occurring between 85 and 163 ms^1^. This deflection is associated with the detection of postural destabilization in response to whole-body perturbations^1^ and may be related to a generalizable aspect of control for initiating an early voluntary postural adjustment^16,17^. Although the cortical localization of the predominant N1 source was found around the supplementary motor area (SMA) in the fronto-central cortex^18^, the generation of perturbation-evoked postural responses is likely governed by a distributed network of sensorimotor sources with frequency-specific topological rearrangement^19^. In this respect, previous studies have proposed that the integration of sensory information into motor commands might be attained by increasing cortical connectivity between prefrontal, (supplementary) motor and posterior parietal areas in temporal association with the perturbation^14,19,20^.

However, in individuals with injuries or impairments somewhere in the sensorimotor system, these mechanisms of functional sensory integration are likely to work insufficiently for regaining postural stability. In elderly people, Parkinson’s disease, patients with traumatic brain injury, peroneal muscular atrophy or chronic lower back pain, for instance, lower amplitudes of the N1 component were shown to be indicative of greater difficulty recovering postural equilibrium^1,21–24^. Moreover, reduced task-related connectivity between middle frontal gyrus, right paracentral lobule, precuneus, and bilateral middle occipital gyri has been linked with poorer postural control^25,26^.

Given that patients after ACLR demonstrate higher functional connectivity in connections (edges) incorporating brain areas (nodes) of the somatosensory and visual cortex when maintaining static postural stability^27^, cortical processing related to the coordination of reactive postural responses may also change as a result of ACL surgery. Characterizing these potential deviations of sensorimotor postural reactions after ACLR could thus provide a more holistic understanding of compensatory strategies accompanying postural instability and help to facilitate tailored rehabilitation protocols for restoring regular postural functions.

Therefore, the aim of the present study was to investigate perturbation-evoked postural and cortical dynamics in patients after ACLR. It was hypothesized that patients after ACLR show reduced postural stability, lower amplitudes of the PEP N1 and concurrently decreased connectivity of sensorimotor edges in response to mechanical perturbations of bipedal stance.

## Results

### Posturography

**Fig. 1:**
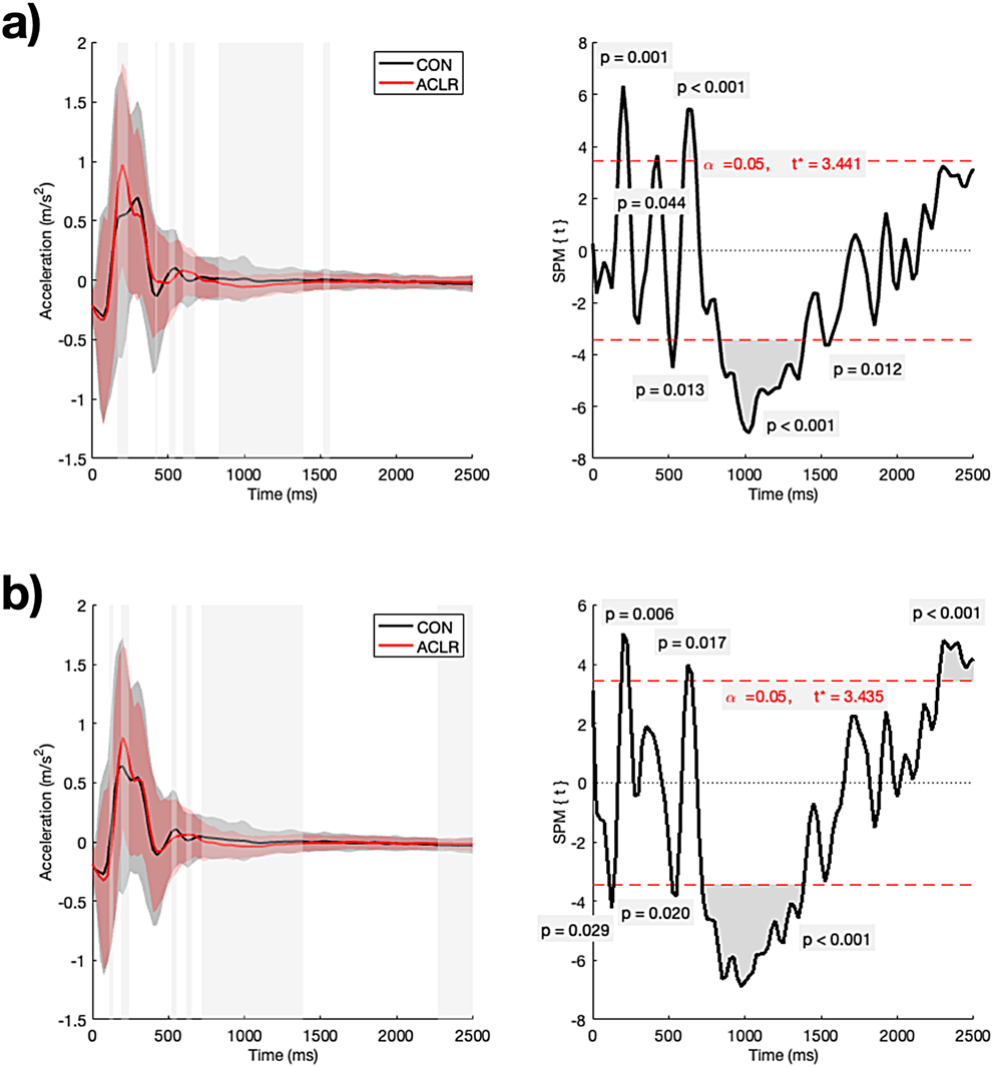
Group differences of anterior-posterior (+/-) acceleration of the hip following perturbation (left panels) and corresponding statistical parametric mapping (SPM{t}) results (right panels) comparing anterior cruciate ligament reconstructed (ACLR, red) and control (CON, black) individuals. **(a)** Differences between individuals included in the perturbation evoked potential (PEP) analysis. **(b)** Differences between individuals included in the effective connectivity analysis. Shaded areas in the left panels represent the corresponding cluster where SPM{t} exceeds the critical significance at α = 0.05 represented by the red dashed horizontal line in the right panels.

SPM analysis of hip acceleration curves between subjects included in the PEP EEG (Fig. 1a) analysis revealed six cluster of data points in which the SPM{t} critical threshold was exceeded. Significant differences between ACLR and CON were found within the time windows of 177–227 ms (*p* = 0.001), 404 to 429 ms (*p* = 0.044), 505–556 ms (*p* = 0.013), 606–682 ms (*p* < 0.001), 833–1389 ms (*p* < 0.001), and from 1515 to 1566 ms *(p* = 0.012).

SPM analysis of differences of hip acceleration between the two groups of subjects included in the EEG connectivity analysis (Fig. 1b) revealed six clusters of data points in which the SPM{t} maximum value exceeded the critical threshold, leading to the rejection of the null hypothesis. The acceleration in the anterior-posterior direction demonstrated significant differences between ACLR and controls within the time windows of 101–126 ms (*p* = 0.029), 177*–*227 ms (*p* = 0.006), 530*–*556 ms (*p* = 0.02), 606–657 ms (*p* = 0.017), 707–1389 ms (*p* < 0.001), and from 2272* to *2500 ms (*p* < 0.001) after the perturbation onset.

### Electroencephalography

**Fig. 2:**
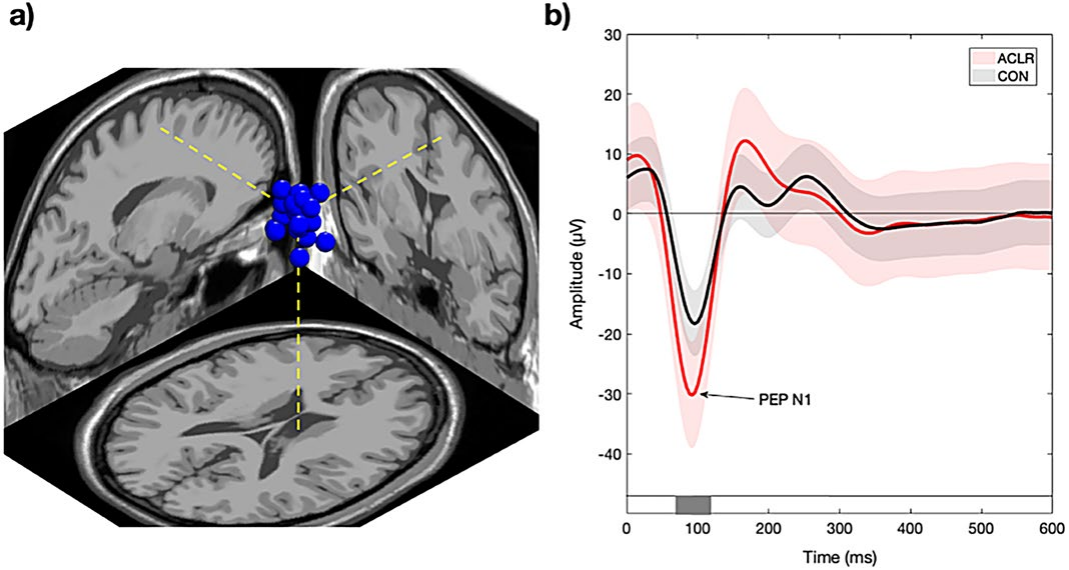
Fronto-central cluster of brain components **(a)** with its corresponding average N1 amplitude of the perturbation-evoked potentials (PEP N1), **(b)** for both the anterior cruciate ligament reconstruction (ACLR, red) and control (CON, black) group. Gray bar represents significant difference at *p* < 0.05.

The cluster of functional brain ICs representing the PEP was initially composed of 26 participants, of which 20 participants (11 ACLR/9 CON) revealed an IC with characteristic ERP polarities, morphology, and peak latency in the fronto-central cortex. In this cluster (Fig. 2a), the PEP N1 amplitude (*T* = −2.708, *p* = 0.017, *CI* [−28.09 −3.299]) was significantly different between the two groups (Fig. 2b).

**Fig. 3:**
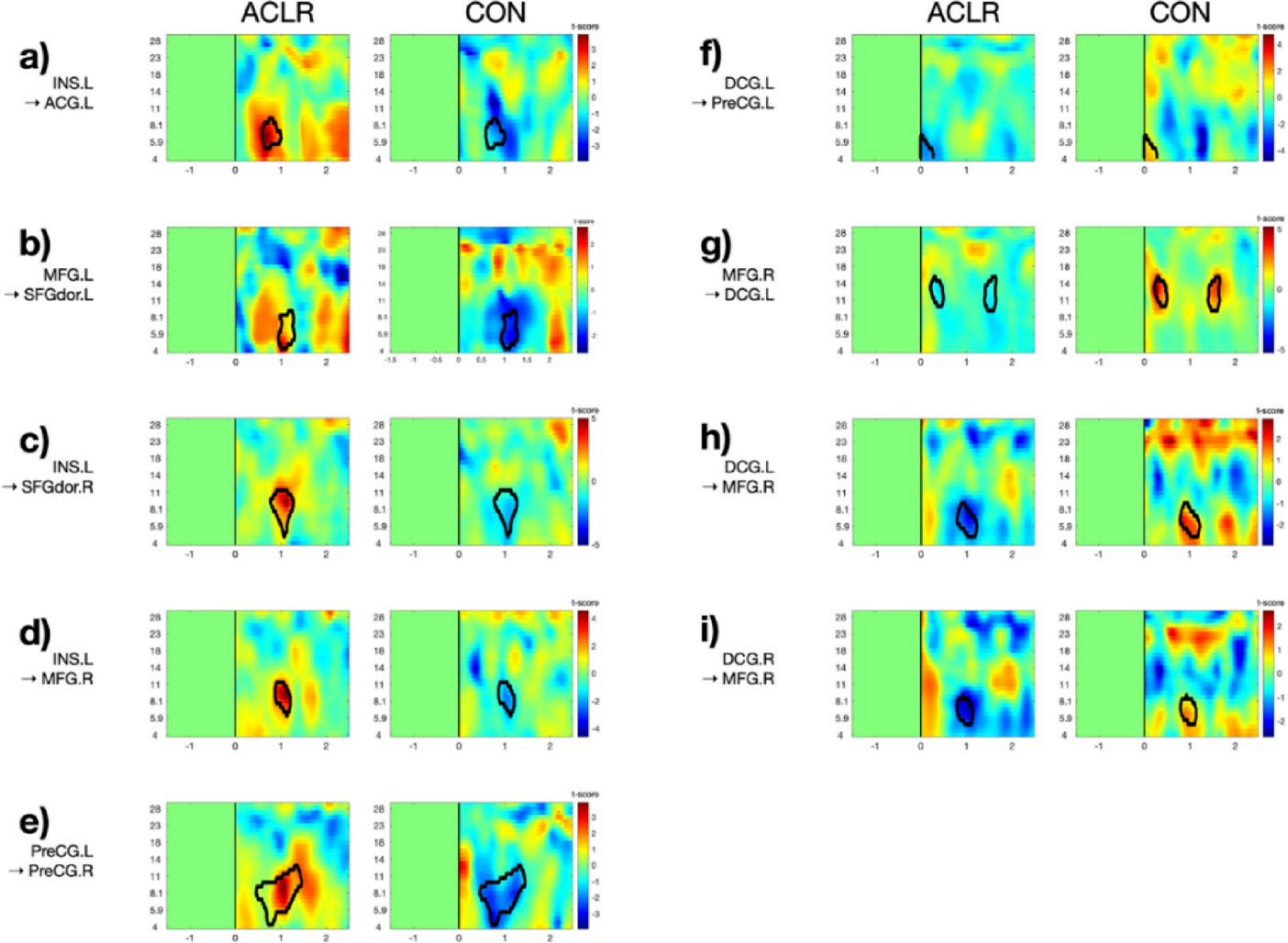
Time-frequency plots of effective connectivity patterns (represented as t-statistics), with time (ms) on the x-axis (with 0 representing the onset of perturbation) and frequency (Hz) on the y-axis, showing significant group differences at *p* < 0.01 (black marking) between anterior cruciate ligament (ACLR) and controls (CON), depicted for estimated significant edges: **(a)** insula left to anterior cingulum left, **(b)** mid frontal left to superior frontal left, **(c)** insula left to superior frontal right, **(d)** insula left to mid frontal right, **(e)** precentral left to precentral right, **(f)** mid cingulum left to precentral left, **(g)** mid frontal right to mid cingulum left, **(h)** mid cingulum left to mid frontal right and **(i)** mid cingulum right to mid frontal right. Abbreviations: *INS.L*, insula left; *ACG.L*, anterior cingulum left, *MFG.L*, mid frontal left; *MFG.R*, mid frontal right; *SFGdor.L*, superior frontal left; *SFGdor.R*, superior frontal right; *PreCG.L*; precentral; *PreCG.R*; precentral right; *DCG.L*, mid cingulum left; *DCG.R*, mid cingulum right.

**Fig. 4:**
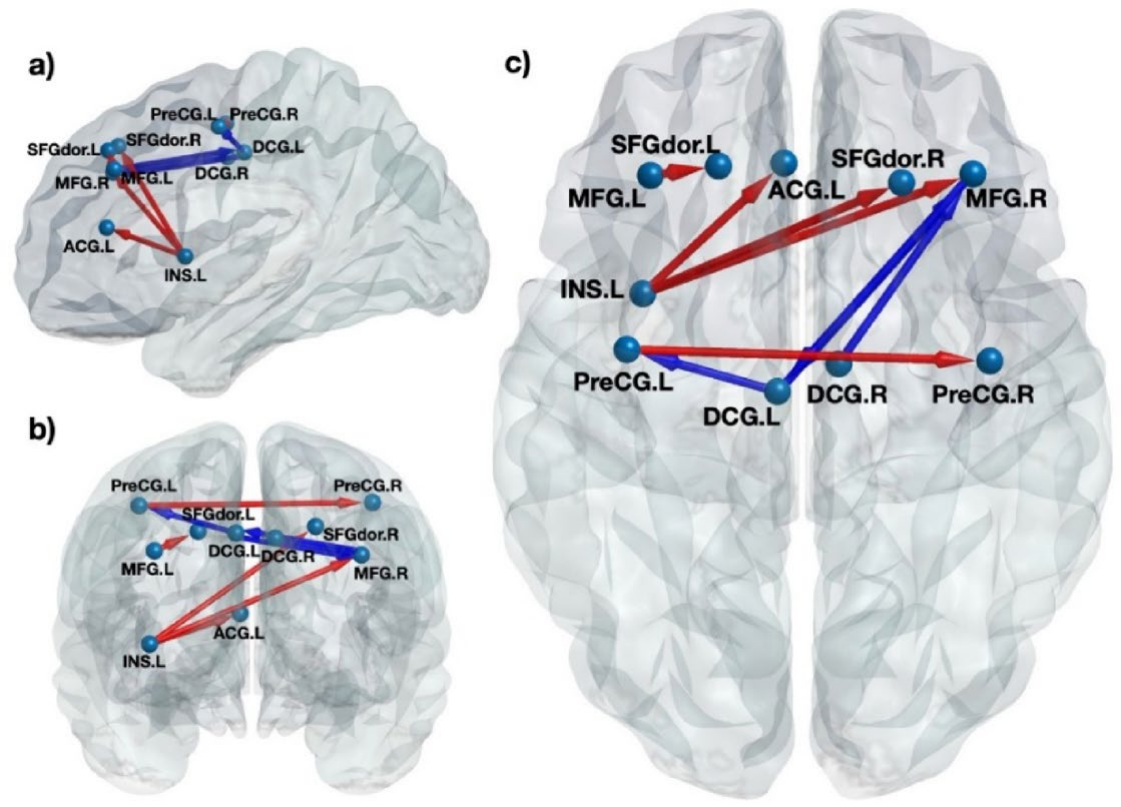
Overview of significant edges between ACLR and CON groups. Arrows represent the direction of information flow, while colors indicate stronger (red) or weaker (blue) effective connectivity in the ACLR group compared to CON. The brain model in **(a)** sagittal, **(b)** coronal and **(c)** axial plane was generated using BrainNet Viewer software^28^. Abbreviations: *INS.L*, insula left; *ACG.L*, anterior cingulum left, *MFG.L*, mid frontal left; *MFG.R*, mid frontal right; *SFGdor.L*, superior frontal left; *SFGdor.R*, superior frontal right; *PreCG.L*; precentral; *PreCG.R*; precentral right; *DCG.L*, mid cingulum left; *DCG.R*, mid cingulum right.

Due to the different approach for group-level comparisons of effective connectivity by utilizing probabilistic dipole densities, 17 ACLR and 13 CON participants were eligible for analysis. Given statistical overlap, the focus of the effective connectivity analyses were placed on 18 AAL graph nodes shared by both groups, of which 9 edges revealed significant group differences at onset (0–200 ms) and predominantly around 1000 ms after the perturbation (Fig. 3). The ACLR group exhibited significantly greater information flow at *p* < 0.01 along edges (Fig. 4) connecting insula left (INS.L) to anterior cingulum left (ACG.L), mid frontal left (MFG.L) to superior frontal left (SFGdor.L), insula left (INS.L) to superior frontal right (SFGdor.R), insula left (INS.L) to mid frontal right (MFG.R), precentral left (PreCG.L) to precentral right (PreCG.R), but significantly lower information flow from mid cingulum left (DCG.L) to precentral left (PreCG.L), mid frontal right (MFG.R) to mid cingulum left (DCG.L), mid cingulum left (DCG.L) to mid frontal right (MFG.R) and mid cingulum right (DCG.R) to mid frontal right (MFG.R).

## Discussion

The aim of the present study was to investigate postural and cortical dynamics elicited by mechanical perturbation to bipedal stance in patients after ACLR compared to asymptomatic controls. The findings revealed that patients after ACLR demonstrated higher acceleration of the hip in the early (~ 200 ms) and later phase (~ 800–1000 ms) following backward platform translations. However, contrary to the initial hypotheses, patients after ACLR showed higher amplitudes of the PEP N1 in response to the external perturbation. Moreover, the ACLR group revealed distinct patterns of effective connectivity as indicated by decreased information flow involving fronto-central edges in the early voluntary adjustment phase, but increased information flow from left to right hemispherical nodes of the frontal cortex at later stages.

When the standing surface is suddenly translated backwards, the hip initially accelerates forward relative to the base of support, as the upper body lags behind the movement of the feet. This forward acceleration of the hips triggers rapid postural responses, typically beginning with activation of the posterior lower leg muscles for initiating a corrective backward acceleration to counteract motion^29^. In the present study, patients after ACLR exhibited significantly stronger acceleration of the hip at 100–230 ms, 750–1400 ms and 2250–2500 ms, while showing significantly resisted hip acceleration around 500–700 ms of the postural response compared to controls. Previous studies have already found that patients after ACLR demonstrate higher sway excursion (~ 200 ms) together with altered muscle activation patterns in response to sagittal plane perturbations^11,12,30^. Consequently, the hip of the ACLR group was displaced further towards the limits of stability, increasing the momentum that must be reversed to restore postural equilibrium in the late phase of the trials^31^.

While the earliest early- and medium-latency postural responses (~ 50–100 ms) after stance disturbances are elicited by monosynaptic spinal circuits, subsequent long-latency voluntary responses (~ 100–200 ms) are mediated by supraspinal centers^32,33^. The characteristic PEP N1 found in this study may thus represent a neural signature of conscious cortical involvement in postural control^1^. This signature may reflect a rapid, modality-independent reset of cortical processing, enabling swift voluntary reactions to sudden postural disturbances^15,16,34^. In that regard, a more salient perturbation is likely to evoke larger brain responses as it demands more immediate attention and potentially urgent corrective motor actions to avoid hazardous situations. Previous studies have already found that increased postural threat enhances the salience of unpredictable perturbations and that this effect is closely linked to subjective posture-related perceptions^35–38^. Adkin and colleagues (2008), for instance, demonstrated that healthy young adults showed increased N1 amplitudes following unpredictable postural perturbations when standing at an elevated threatening height (3.2 m) compared to ground level. Moreover, this augmentation of the N1 was correlated with psychological scores for fear of falling, anxiety, reduced confidence and lower perceived postural stability. Complementary, Spieser et al. (2010) reported that intentional processes may modulate early cortical responses to mechanical perturbations during voluntary movement. Specifically, when subjects intended to actively ‘resist’ rather than ‘let-go’ sensory perturbations, the amplitude of the perturbation-evoked N1 was significantly enhanced^39^. Fear of re-injury has been shown to substantially affect motor performance in patients after ACLR^40,41^. As descriptive KOOS scores indicated functional impairments, potentially harmful postural destabilization might be attributed greater salience in the present ACLR sample. Consequently, more voluntary control might be a strategy for resisting destabilizing external forces in these individuals^35–37,42,43^.

Interestingly, while the central cluster for generating the PEP N1 - with its characteristic location in proximity to the SMA – indicated a stronger neural response in patients after ACLR, effective connectivity linking this area with other nodes in the frontal cortex decreased bidirectionally. These differences of causal interactions between groups were most present in the theta frequency range and aligned temporarily with stronger hip acceleration in the ACLR group (~ 100–200 ms/~1000 ms after perturbation onset). Earlier research has demonstrated that postural adjustments are modulated by the activity of theta oscillations within a frontal network, which facilitate the integration of sensory information as well as the initiation of reactive postural responses^42,44–46^. Additionally, the theta frequency range in motor areas of the brain has been attributed an important role in motor control during both the initiation and termination phases of a postural response^20,47^. Thus, stronger N1 amplitudes alongside reduced information flow in fronto-central motor regions during the long-latency postural responses may suggest that postural perturbations evoke greater cortical alert in ACLR patients for presumably voluntarily resisting external destabilization. However, despite this increased alertness, the integration of sensorimotor information appears to be compromised and still results in sub-optimal postural responses.

Contrary to the decrease in fronto-central effective connectivity observed in the ACLR group, the analyses also revealed a significant augmentation of causal information flow in theta and alpha frequency ranges converged on nodes across prefrontal, insular and precentral motor areas in the ACLR group. These differences of information flow were primarily related to edges directed towards the right hemisphere in the late re-stabilization following the perturbations. Findings by Duclos et al. (2015)^48^ and Fernandes et al. (2018)^49^ have suggested a potentially crucial involvement of the right hemisphere in postural control. These investigations found that lesions to the right hemisphere of the brain were associated with considerably more pronounced postural impairments in patients with stroke than lesions to the left hemisphere. According to the authors, the right hemisphere may thus contribute more substantially to the efferent processes governing the regulation of upright posture. Moreover, the right cerebral hemisphere was proposed to exert a specialized function in the active regulation of limb stiffness. This function enhances movement stability during the final phase of joint motion by mediating rapid sensorimotor feedback processes and thereby facilitating postural stability when counteracting mechanical perturbations^50,51^. Initial braking of the hip acceleration appeared to be less effective and led to more postural instability in the ACLR group. As a result, the requirement for corrective action at later stages of the postural response intensifies. The observed increase in frontal interhemispheric information flow in the theta and alpha frequency band may thus serve as a compensatory voluntary strategy to mitigate postural instability during re-stabilization in the ACLR group^27,44,52^.

Lastly, it should be noted that although the late between-group differences in hip acceleration (~ 1000ms) were small in mechanical terms and are thus unlikely, in isolation, to reflect clinically meaningful functional deficits, their significance may lie in their association with the observed neural responses. Specifically, the increased connectivity during the late stabilization phase may indicate that individuals after ACLR may continue to recruit cortical control processes beyond the time window in which asymptomatic controls have largely regained stable posture. From a functional perspective, such findings are consistent with a less automatic and potentially more resource-dependent mode of postural control^27,53^, whereby apparently subtle biomechanical differences may conceal persistent alterations in the neural strategies supporting postural stabilization. In line with previous observations in unperturbed single leg stance^27^, the present findings may suggest that patients after ACLR who rely on voluntary sensorimotor control strategies - instead of automatic postural processes - might not have attained full functional recovery. This shift towards more conscious, less efficient sensorimotor control strategies may increase cognitive load and reduce movement adaptability in extremely dynamic situations, thereby elevating the risk for postural misalignment and re-injury. Consequently, rehabilitation may prioritize tailored exercises in multisensory dynamic environments, particularly perturbation-based interventions designed to enhance automatic postural responses. Such approaches may help restore efficient postural control and reduce reliance on voluntary control.

## Methodological considerations

In the present study, a mobile brain and body imaging approach was employed to examine perturbation-evoked postural and cortical responses in patients after ACLR. Nonetheless, it is important to recognize several methodological limitations that may influence the interpretation of these results. First of all, while many investigations of postural control in patients after ACLR solely included subjects with isolated tears and homogeneous graft types, the experimental group in the present study consisted of a relatively heterogenous sample. Similar to the ACL, menisci contain mechanoreceptors and are innervated by the posterior articular branch of the tibial nerve^54^. Consequently, meniscal tears have been reported to cause persistent proprioceptive deficits despite clinically successful surgical repairs^55^. Nevertheless, Park and colleagues (2015) reported no significant side-to-side differences in postural instability between the injured and uninjured limbs in patients with either isolated ACL tears or ACL tears accompanied by medial meniscus injury^56^. Whereas evidence is lacking regarding the effects of meniscal injury on sensorimotor processing, cortical contributions to postural control may appear differently in ACLR patients with concomitant meniscal tears. In line with this, the relatively large variability in time from injury to surgery and in postoperative testing time, but also therapeutical treatment may also have influenced the observed outcomes. Although previous studies suggested a potential effect of injury-/surgery-specific factors on sensorimotor control^53,57–59^, the general body of available evidence still lacks sufficient control of critical variables such as time since injury. Given the modest sample size, the present study was not adequately powered to perform covariate or subgroup analyses without a substantial risk of unreliable estimates. Future approaches may therefore explicitly investigate the influence of various factors such as concomitant injury, graft type or leg dominance on cortical processing related to postural control in patients after ACLR.

Second, while averaging event-related brain dynamics across multiple trials will reduce random noise and thereby improving the reliability of the extracted neural features^1^, the large number of repeated perturbations and anticipated direction of the translation may impose habituation which could affect the behavioral and cortical observations. Lysholm and colleagues (1998), for instance, reported that the initial platform-induced perturbation elicited significantly greater postural instability compared to subsequent perturbations in both ACLR and control participants. Although this effect could not be excluded in the present study, the experimental protocol allowed to distinguish the two groups based on their behavioral and cortical dynamics.

Additionally, several methodological aspects of the employed EEG data processing pipeline may potentially have modulated the observed results. First, with reference to the inverse problem, the ICA source model with its equivalent dipole locations inherently represents an approximation of the underlying cortical dynamics. Therefore, the spatial assignment of electrical activities should inherently be considered with caution. Moreover, the prioritization of consistently identified ICs across participants may have excluded cortical sources engaged in sensorimotor processing pertinent to reactive postural control, potentially yielding an incomplete picture of brain network interactions. As the stability of MVAR models depends on similar input parameters per subject^60^, subject-specific IC selection was constrained to the top seven ICs explaining the greatest variance. However, considering the presumed topographical organization of functional networks subserving reactive postural control^44^, this selection strategy may have overlooked other subject-specific sources. Second, the application of ICA for source localization inherently assumes statistical independence between underlying sources, a premise that theoretically contrasts with the potential for causal relationships between ICs. While ICA decomposition relies on instantaneous dependencies within time series data, rPDC – as a metric of effective connectivity – infers causality based on the influence of past activity on current states. Although conceptually distinct, these approaches are not necessarily mutually conflicting^61^. Notwithstanding, the validity of inferred effective connectivity is predicated on the establishment of a stable MVAR model, ideally satisfying criteria of whiteness, consistency and stability to mitigate the risk of overfitting^62^. In this regard, third, the analysis revealed that the residuals from the fitted MVAR model marginally failed the autocorrelation function whiteness test (*p* = 0.91/0.92 < 0.95), suggesting a suboptimal representation of the underlying data structure. These minor deviations from white noise properties within the residuals could indicate the presence of unmodeled variables, model misspecification, or other factors compromising the reliability of model outcomes^61^. However, the parameter-to-data point ratio (0.08 < 0.1), data consistency metrics (> 75%), and model stability assessments (−0.08 < 0) demonstrated adherence to accepted standards for appropriate model fit and likely enabled the model to account for a substantial portion of the observed neurophysiological dynamics during the postural perturbations^61,63^.

## Conclusion

In conclusion, individuals after ACLR exhibited less efficient motor behavior in response to sudden mechanically-evoked postural instability, whereas the generation of the perturbation-evoked postural responses was likely influenced by alterations of frequency-specific causal information flow in a distributed network of fronto-central nodes. This may imply that patients after ACLR face heightened perceived threat or increased voluntary effort to resist potentially harmful postural instability, ultimately leading to increased cortical alertness and subsequently less automatic sensorimotor processing in response to external sensory disturbances. Consequently, more consciously regulated and less efficient postural adjustments - particularly with respect to the coordination of individual joints - may predispose individuals after ACLR to an elevated risk of re-injury. Thus, further research is needed to delineate the specific neural mechanisms underlying altered postural control strategies after ACLR and to determine whether targeted interventions might be able to promote more automatic and efficient sensorimotor integration in this population.

## Methods

### Subjects

Seventeen athletes (8 female/9 male; age: 23.6 ± 3.7 years; height: 176.9 ± 10.2 cm; weight 76.2 ± 13.4 kg, Table 1) who had undergone anterior cruciate ligament reconstruction (ACLR, 8 right/9 left), completed their rehabilitation program, and subsequently returned to their pre-injury sport were recruited for this study. Participants underwent ACLR surgery on average 61.0 ± 56.9 days after injury and were assessed 9.4 ± 5.2 months after surgery for this study. A patellofemoral tendon was used as the graft material for ligament reconstruction in four participants, whereas a semitendinosus tendon was utilized in twelve participants. One participant could not provide information regarding the graft used. Of the ACLR participants included, only one participant reported concomitant cartilage damage. Eight participants reported concomitant meniscectomy or meniscus repair, three of whom reported an associated medial collateral ligament injury, and one participant reported a lateral collateral ligament injury (Table 2). The study excluded participants who fell outside of the age range of 18 to 40 years, those who disclosed neurological or psychological disorders, severe musculoskeletal injuries in the lower limbs, or were taking neuroactive or psychoactive medications. A control group of fifteen asymptomatic individuals was recruited according to weekly physical activity and anthropometric characteristics similar to those of the ACLR group. Two initially recruited controls were excluded, either due to insufficient EEG data quality or irregular sway pattern, resulting in a final control group of thirteen participants (4 female/9 male; 25.9 ± 4.2 years; 178.5 ± 8.8 cm; 75.1 ± 12.1 kg, Table 1). All participants gave written consent prior to the measurement process. The present study procedures were designed in line with the declaration of Helsinki and received ethical approval from the ethics committee of Paderborn University.

**Table 1:** Descriptive statistics of the ACLR and CON groups.

	ACLR	CON	*p* - value
*Demographics*			
Sex (female/male)	8/9	4/9	
Age (years)	23.6 ± 3.7	25.9 ± 4.2	0.126
Height (cm)	176.9 ± 10.2	178.5 ± 8.8	0.657
Weight (kg)	76.2 ± 13.4	75.1 ± 12.1	0.737
Dominant stance leg (left/right)	11/6	7/6	
*Physical activity*			
Sporting activity (years)	11.4 ± 6.1	16.5 ± 8.6	0.039*
Times active per week (days)	3.2 ± 1.2	2.6 ± 0.7	0.226
*KOOS score*			
Pain (%)	87.7 ± 8.5	99.4 ± 1.2	0.001*
Symptom (%)	73.7 ± 15.4	94.1 ± 11.6	0.001*
Activities of daily living (%)	95.5 ± 5.6	99.9 ± 0.3	0.001*
Sport/recreation (%)	77.1 ± 21.4	99.3 ± 2.7	0.001*
Quality of life (%)	59.2 ± 19.0	97.8 ± 8.4	0.001*
*IKDC score*			
Total score (%)	79.7 ± 12.9	99.6 ± 1.1	0.001*
Knee function (pre-surgery, scale 0–10)	9.7 ± 0.6	9.92 ± 0.3	0.171
Knee function (post-surgery, scale 0–10)	7.8 ± 1.7^Δ^	-	0.001*

ACLR: patients after anterior cruciate ligament reconstruction; CON: controls; KOOS: Knee Injury and Osteoarthritis Outcome Questionnaire; IKDC: International Knee Documentation Committee Questionnaire; *significant difference between groups at *p* < .05; ^D^significant difference within ACLR group at *p* < .001.

### Procedure

**Fig. 5:**
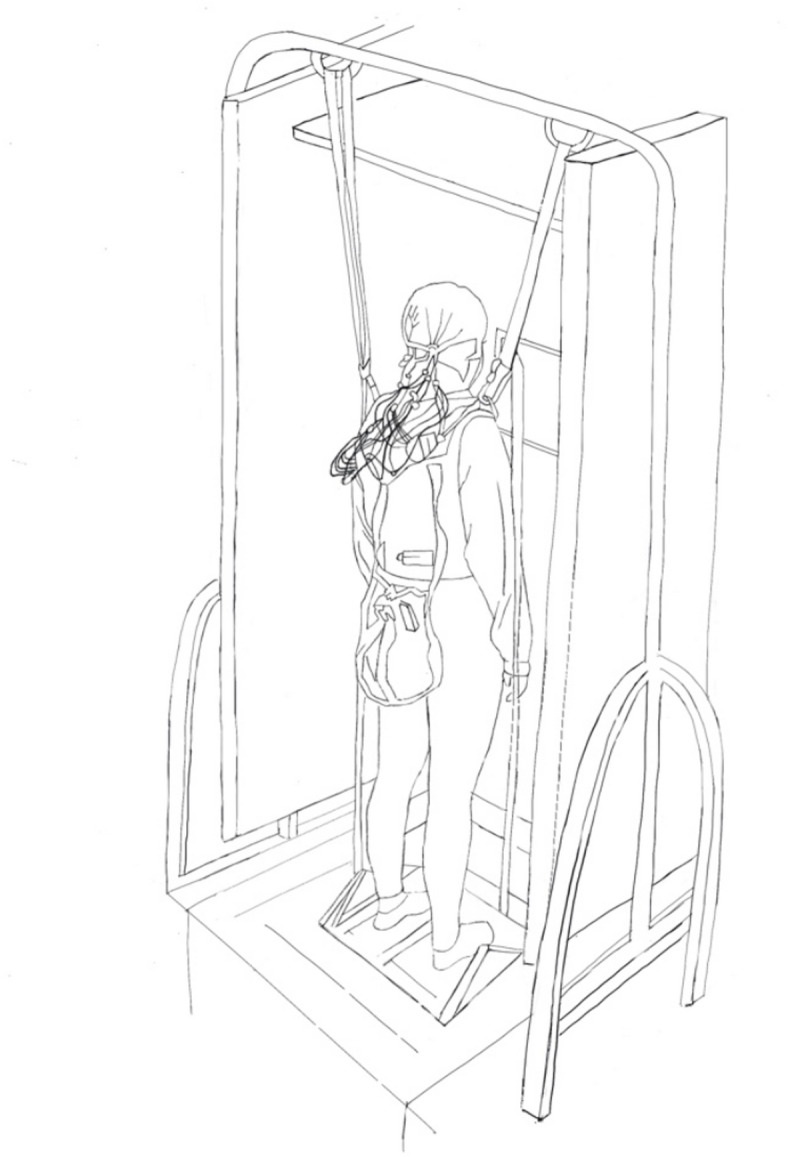
Visualization of experimental configuration with a subject inside the NeuroCom^®^ device.

Upon arrival, information regarding the experimental procedure, the rights of the subjects and the possible risks of being part of the current study were provided. Subsequently, the subjects gave written consent and proceeded to fill out anamnesis and knee functionality questionnaires. The process then proceeded with the application and fitting of the EEG cap. After completing the EEG preparation, participants were asked to enter the NeuroCom^®^ SMART EquiTest^®^ (NeuroCom Int., Inc., Clackamas, Oregon) for evaluating their postural stability during backward surface translations. Participants stood barefoot on a moving force plate platform within the NeuroCom^®^ with arms parallel to their torso, whilst wearing a safety harness to avoid any falls (Fig. 5).

Each perturbation trial involved the platform moving backwards (amplitude of 4.064 cm, duration of 256 ms, ramp delay of 1500 ms), followed by a gradual forward movement to its starting position. Backward platform translations were chosen based on the assumption that they descriptively induce greater postural sway than forward translations in both ACLR and healthy individuals^12^. Although the general characteristics of PEPs, including polarity, morphology, and onset/peak latency, appear to be similar across perturbation directions and support conditions in healthy participants^38,64,65^, the N1 amplitude has been shown to increase with perturbation salience^35,37,39^. Backward perturbations were therefore expected to provide a stronger yet methodologically comparable stimulus, which may enhance the ability to detect potential differences between groups. Although participants were informed about the direction of the platform translations, neither information about the intensity nor the timing were provided. The examiner manually started each trial sequence, which led to naturally randomized perturbation onsets. Each participant completed 100 trials in total, consisting of five blocks of 20 trials each. A one-minute break, in which participants stayed in the NeuroCom^®^ with the freedom to move, was given after each block. Moreover, a five-minute break after the fifth block was provided to allow participants to step out of the machine and to prevent physical fatigue. If required, a maximum five-minute break was offered after a block owing to exhaustion or discomfort. Participants were instructed to remain stable during the perturbation phase, with relaxed facial muscles and a steady head position, looking straight ahead.

**Table 2:**
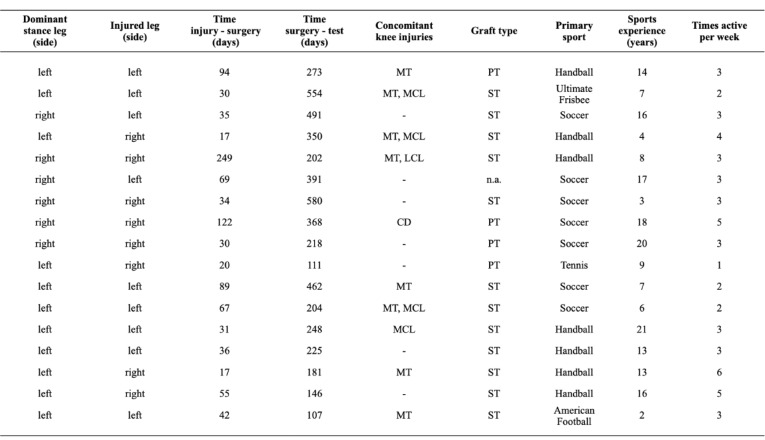
Injury- and sports-related characteristics of patients with anterior cruciate ligament reconstruction.

MT, meniscus tear; MCL, medial collateral ligament rupture; LCL, lateral collateral ligament rupture; CD, cartilage damage; PT, patella autograft; ST, semitendinosus autograft.

### Knee function evaluation

Knee functional ability was evaluated based on a self-reported survey: the Knee Injury and Osteoarthritis Outcome Questionnaire (KOOS,^66^). The KOOS survey consists of five dimensions, including pain (nine items), symptoms (seven items), daily living function (17 items), sport and recreation function (five items), and knee-related quality of life (four items). The KOOS score (Table 1) was calculated by summing the scores assigned to each item within its dimension, with each item receiving a score between 0 and 4. For each subscale, the raw sum was then transformed to a 0–100 scale, where 0 indicates extreme problems and 100 indicates no problems.

### Accelerometry

In order to determine the onset of the perturbation-induced body movement, a hip belt was used to place a triaxial accelerometer sensor integrated in the EEG amplifier (LiveAmp64, Brain Products, Germany) on the sacrum of participants. Accelerometry data was recorded in three directions (x, y, z) at a sampling rate of 500 Hz. For the offline processing, a custom-built detection algorithm (R2022b, Mathworks Inc, Natick, USA) was used for determining the start of hip acceleration. The algorithm incorporated data smoothing using Gaussian-weighted moving average over a window of 150 samples. The onsets of the platform perturbation were defined as the first linear change (*findchangepts* function, *MaxNumChanges* = 8) in signal mean and slope surrounding local maxima (*findpeaks* function), which were filtered by their intrinsic height (*MinPeakHeight* = standard deviation of signal) and relative peak-to-peak separation^67^. The resulting timepoints were used for the subsequent epoching of the EEG data and accelerometer signal for further analysis.

### Postural stability assessment

A custom-built MATLAB (R2024b, Mathworks Inc, Natick, USA) script was used to process and analyze the extracted acceleration signal after the perturbation onset. Given the nature of the perturbation and the accelerometer placement, the analyses were solely based on the anterior-posterior component of the acceleration signal. Signal processing involved the removal of the gravity component by subtracting from the original signal the corresponding low-pass filtered signal using a first-order Butterworth filter with a cutoff of 0.35 Hz^68^. Subsequently, signal noise was removed by applying a second-order Butterworth low-pass filter, with a cutoff of 10 Hz, and data were downsampled at 100 Hz. In line with EEG analysis requirements, the accelerometer data of the first EEG 50 artifact-free trials were considered for the subsequent statistical analysis.

### Electroencephalography

Brain activity was recorded using 65 active Ag/AgCl electrodes (actiCap, Brain Products, Munich, Germany) placed according to the extended international 10–20 system and transmitted through a wireless transmission path (LiveAmp64, Brain Products, Munich, Germany). The EEG signals were digitally amplified at a sampling rate of 500 Hz and online low-pass filtered at 200 Hz. The amplifier was placed in a hip belt at the level of the sacrum to allow unrestricted mobility. In order to ensure an appropriate signal-to-noise ratio, electrode impedance was kept below 25 kΩ, as recommended by the manufacturer. Furthermore, an online FCz reference montage was used, including AFz as the ground electrode.

EEG data processing was performed in EEGLAB open source toolbox^69^ for MATLAB. Initially, the FCz reference channel was restored and the perturbation onsets detected from the accelerometer timeseries were added to the EEG event structure. Afterwards, sinusoidal line noise (50/100 Hz) was removed using *Cleanline*^70^ and a basic finite impulse response filter with a band pass of 3–30 Hz was applied. The data were re-referenced to common average and downsampled to 250 Hz. Additionally, *clean_rawdata*^71^ was used to remove channels with transient or high amplitude noise (line noise criterion: 4), poor correlation with adjacent channels (channel criterion: 0.8), prolonged flatline channels (flatline criterion: 5) and to apply automatic subspace reconstruction for removing and interpolating non-stationary high-amplitude bursts with large variance (burst criterion: 10). In the case of channel rejections, the data were re-referenced to common average again. For the quantification of cortical network dynamics, the continuous EEG data was divided into epochs ranging from − 2000 ms to 3000 ms relative to perturbation onset.

To decompose the data into maximally independent components (ICs) of electrophysiological activity, adaptive mixture independent component analysis^72^ was applied to the pre-processed scalp recordings. A default MNI head model of the DIPFIT function^73^ was then used to estimate equivalent dipole locations of the decomposed sources. The set of ICs was then classified based on their individual scalp topography, equivalent dipole location and activity power spectrum^74^. In order to achieve a robust statistical model, each subject was restricted to their top 7 ICs (minimum number of functional ICs across participants) based on variance accounted for. Subsequently performed k-means clustering was then used to aggregate PEP ICs based on common spatial features. The cluster was selected with respect to centroid location in proximity to the SMA, as this cortical region has previously been associated with the PEP N1^18^. Only participants exhibiting a distinct IC with characteristic polarity, morphology, and peak latency consistent with a typical PEP were included in further analysis.

The N1 amplitude was computed by first identifying the dominant negative peak of the PEP time series following the onset of the perturbation. The process involved segmenting the data epochs around the perturbation event with a pre-perturbation baseline period from − 2000 ms to 0 ms. After averaging all trial ERPs for each individual subject, the N1 amplitude of the mean PEP was defined as the peak negative voltage within the 0–200 ms latency after perturbation onset.

For the analysis of event-related causal information flow between the multivariate IC time series, renormalized partial directed coherence (rPDC) was calculated across the sources, including brain oscillations in theta (4–7 Hz), alpha (8–12 Hz) and beta (13–30 Hz) frequency ranges^75^. A parametric multivariate autoregressive (MVAR) model was applied to the ICA-derived sources of sensorimotor brain activity utilizing *groupSIFT*^60,76^. To fit the MVAR model for each dataset with respective ICs for each group (ACLR/CON), a grand-average optimum model order of 10, a sliding Hamming window with a length of 1.0 s and a step size of 0.02 s was selected, finally generating 30 log-scaled frequency bins from 4 to 30 Hz. The model for both groups was then validated based on various metrics, including model order (8.35 ± 0.61/8.46 ± 0.52), statistical whiteness (0.91 ± 0.02/0.92 ± 0.01), data consistency (75.62 ± 2.57/76.13 ± 1.48), parameter-to-data point ratio (0.08 ± 0.01), and model stability index (−0.08 ± 0.01).

Previously estimated dipole locations were converted into probabilistic dipole densities using a 3D Gaussian kernel with a full-width at half maximum of 20 mm, truncated at 3 sigma, to address spatial variability between participants. This transformation allowed for the conversion to group anatomical regions of interest (ROI) based on the automated anatomical label atlas (AAL,^77^), requiring at least 70% of participants to contribute non-zero dipole densities^60^. As a result, 18 of the original 76 AAL graph nodes exhibited overlap across both groups (ACLR/CON), leading to the creation of a connectivity matrix that depicted differences of causal information flow based on ROI-to-ROI pairwise dipole densities weighted by rPDC.

### Statistical analysis

All statistical analysis were conducted in MATLAB (R2022b, Mathworks Inc, Natick, USA) and statistical outliers were removed when exceeding three scaled median absolute deviations from the median using built-in *isoutlier*-function. Statistical parametric mapping (SPM) was used to compare the acceleration of the hip in the anterior-posterior direction between the two groups. Specifically, SPM two-tailed independent t-test was used (α = 0.05). SPM analysis was conducted using the SPM1D (v.04.10) open-source code^78^.

Peak amplitudes of the N1 component were tested for normality using the Shapiro-Wilk test and then compared between the two groups using two-sample permutation-test based on Welch’s t-statistic with 10,000 iterations. The corresponding *p*-values were adjusted for multiple comparisons using the maximum correction method^79^.

Source-level connectivity statistics were computed using the *groupSIFT* framework previously described. For each graph edge, pixelwise two-sample t-tests were conducted on the rPDC time-frequency maps between the two groups, applying an initial uncorrected threshold of *p* < 0.01. Clusters of adjacent pixels with significant t-statistics were identified, highlighting significant differences between conditions in the time-frequency domain. The t-score maps were subsequently corrected for multiple comparisons using permutation tests with 10,000 iterations at *p* < 0.01 and generalized family-wise error rate control for cluster-level correction^80,81^.

## Data Availability

The datasets generated during and/or analyzed during the current study are available from the corresponding author on reasonable request.
